# Implementation of a difficult airway rapid response team: a Brazilian experience

**DOI:** 10.1016/j.bjane.2026.844741

**Published:** 2026-03-06

**Authors:** Bernard Marcel Barban, Verônica Neves Fialho Queiroz, Ayrton Bentes Teixeira, Daniel Sousa Cesar, Flavia Baldavira Hirano, Michele Jaures, Waldyr Muniz Oliva Neto, Roseny dos Reis Rodrigues, Maria José Carvalho Carmona

**Affiliations:** a Hospital Israelita Albert Einstein, Department of Anesthesiology, São Paulo, SP, Brazil; b Hospital Israelita Albert Einstein, Department of Care Practice, São Paulo, SP, Brazil; c Integrated Hospital Care Unit, Anesthesiology Department, Abu Dhabi, United Arab Emirates; d Intensive Care Departament from Albert Einstein Hospital São Paulo, SP, Brazil; e Faculdade de Medicina da Universidade de São Paulo, Discipline of Anesthesiology, São Paulo, SP, Brazil

*Dear Editor*,

Failure to obtain a secure airway can escalate within minutes to hypoxemia, cardiac arrest, and devastating neurological injury. Although specialty guidelines provide robust algorithms for managing difficult airways, real-world hospital care often fails at critical interfaces ‒ namely, early recognition, rapid mobilization, and coordinated execution across diverse clinical settings. Motivated by this gap and by international experiences, we designed and implemented a hospital-wide Difficult Airway Rapid Response Team (DART) at Einstein Hospital Israelita, a large private tertiary general hospital in São Paulo, Brazil. The institution features modern infrastructure, 647 beds (including 149 ICU beds), and a high-volume emergency department. We also developed a pragmatic manual to support adoption in similar institutions.

Our program is modeled after the Johns Hopkins DART (Difficult Airway Response Team). This multidisciplinary program was created to improve the emergency management of difficult airways outside the operating room. DART brings together specialists from anesthesiology, emergency medicine, otolaryngology, and trauma surgery, responding through a rapid paging system to critical airway events. The team is equipped with advanced airway carts and follows strict activation criteria, including known or encountered difficult airways that standard code teams are unable to manage. The Johns Hopkins DART program was implemented after root cause analyses identified safety gaps in airway emergencies and includes operational protocols, equipment management, and extensive simulation-based training. Since 2005, this model has enhanced patient safety, ensured more reliable emergency responses, and substantially reduced airway-related adverse events and the need for surgical airways in non-operative hospital settings.^1^

During the COVID-19 pandemic, DARTs were essential for adapting hospital airway management protocols to the increasing numbers of critically ill patients and heightened infection risks for healthcare professionals. These expanded teams, composed mainly of anesthesiologists and ICU specialists, followed strict protocols to maximize intubation success, consistently used Personal Protective Equipment (PPE), and minimized mask ventilation to reduce aerosolization risks. Hospitals redeployed experienced professionals to form rapid airway response teams and implemented standardized processes emphasizing effective communication and equipment preparedness. Healthcare worker protection was prioritized through focused training, exclusion of high-risk staff, and rigorous hygiene practices. This multidisciplinary model improved care coordination and contributed to reducing adverse airway events, making these teams a key component of hospital responses during the COVID-19 outbreak.^2^

Our manual incorporates evidence-based clinical pathways supported by systems engineering principles. Activation criteria were standardized and include: (i) Anticipated difficult airway as judged by the attending clinician; (ii) Persistent hypoxemia during airway management (SpO_2_ < 90%); (iii) More than two unsuccessful intubation attempts; or (iv) Tracheostomy displacement. We established a single dedicated call number, set an arrival target of ≤ 5-minutes, and ensured hospital-wide coverage across all clinical areas, including ICUs, step-down units, the emergency department, operating rooms, obstetrics, diagnostic units, wards, and nurseries.

The pediatric difficult airway response follows a workflow similar to the adult model. Primary management is performed by the anesthesiologist; however, second-line backup differs, utilizing an adult intensivist for adults and a pediatric intensivist for children. Any healthcare professional involved in patient care may activate the DART. Upon activation, all on-duty members of the multidisciplinary response team are paged and immediately proceed to the event location.

Operational readiness relies on three pillars: (1) Prepositioned adult and pediatric difficult-airway kits available on every floor and sector; the local team retrieves the kit and brings it to the scene. Each kit contains a checklist of items periodically restocked and checked by the pharmacy team and verified by the sector nursing staff to ensure the availability of all necessary devices in all sizes (videolaryngoscopes, supraglottic devices, bougies/guides, capnography, and surgical airway sets). (2) A clear role map at the scene (unit team lead, anesthesiology lead, anesthesia-practice nurse, and respiratory/physiotherapy support; critical-care physicians act as second-line leaders during off-hours). (3) A concise, mandatory handoff and capnography confirmation of airway placement. Each event concludes with a 2–5 minute debrief and the completion of a structured electronic record to enable process control.

Training is the cornerstone of the program. We implemented structured onboarding and biannual refresher sessions using skill stations and high-fidelity simulations of high-stress scenarios, including anticipated and unanticipated difficult airways, “Cannot-Intubate/Cannot-Oxygenate” (CICO) situations, and tracheostomy emergencies. Program governance is provided by a multidisciplinary committee (Anesthesiology, Critical Care, Emergency, Nursing, Respiratory Therapy, and Quality/Safety), which oversees audits and continuous improvement using predefined indicators: response time, first-pass success, severe hypoxemia, need for surgical airway, complications, and documentation completeness. These metrics are tracked via a dashboard by hospital leadership.

Implementation revealed several challenges, including adherence to activation criteria, after-hours anesthesiology coverage, intra-hospital transit delays, and incomplete documentation. Mitigation strategies included empowering nursing staff to activate the team, designating intensivists as second-line leaders overnight, prioritizing elevators and transport workflows, and implementing real-time audits with clinician feedback. These countermeasures mirror lessons reported by mature international programs and proved feasible in our context.

Beyond algorithms, our experience highlights that difficult airway safety depends on institutional design, encompassing rapid detection, reliable mobilization, clearly defined roles, standardized equipment, simulation-driven competency, and continuous measurement. We therefore share a concise implementation manual covering governance, activation criteria, call and escalation logistics, equipment checklists, scene roles, stepwise clinical plans (Plan A/B/C with mandatory capnography), documentation templates, training curriculum, and performance targets. The manual is intentionally adaptable to the resource variability common across Brazilian hospitals.^3^^,^^4^

Limitations of our report include its single-center nature and the absence, to date, of time-series outcome analysis. We are initiating a before-and-after evaluation and invite collaboration toward a multicenter Brazilian registry focused on process and clinical outcomes (e.g., first-pass success, surgical airway rates, and mortality). Ethics committee approval was waived for this letter due to its retrospective and non-interventional nature.

In summary, a hospital-wide difficult airway rapid response team is feasible and effective when supported by clear activation criteria, rapid mobilization, standardized equipment, simulation-based training, and rigorous governance.

The complete implementation manual is available at the following link in the anesthesia section: https://medicalsuite.einstein.br/pratica-medica/SitePages/pathways.aspx.

## Data availability statement

No new data were created or analyzed in this study. Data sharing is not applicable to this article.

## Conflicts of interest

The authors declare no conflicts of interest.
